# Photosynthetic temperature responses in leaves and canopies: why temperature optima may disagree at different scales

**DOI:** 10.1093/treephys/tpae135

**Published:** 2024-10-17

**Authors:** Dushan P Kumarathunge, Belinda E Medlyn, John E Drake, Martin G De Kauwe, Mark G Tjoelker, Michael J Aspinwall, Craig V M Barton, Courtney E Campany, Kristine Y Crous, Jinyan Yang, Mingkai Jiang

**Affiliations:** Hawkesbury Institute for the Environment, Western Sydney University, Locked Bag 1797, Penrith, NSW 2751, Australia; Department of Agricultural Biology, Faculty of Agriculture, University of Ruhuna, Mapalana, Kamburupitiya, Kamburupitiya 81100, Sri Lanka; Hawkesbury Institute for the Environment, Western Sydney University, Locked Bag 1797, Penrith, NSW 2751, Australia; Hawkesbury Institute for the Environment, Western Sydney University, Locked Bag 1797, Penrith, NSW 2751, Australia; Department of Sustainable Resources Management, College of Environmental Science and Forestry, State University of New York, 1 Forestry Drive, Syracuse, NY 13210, USA; School of Biological Sciences, University of Bristol, Bristol BS8 1TQ, UK; Hawkesbury Institute for the Environment, Western Sydney University, Locked Bag 1797, Penrith, NSW 2751, Australia; Hawkesbury Institute for the Environment, Western Sydney University, Locked Bag 1797, Penrith, NSW 2751, Australia; Formation Environmental, LLC, Sacramento, CA 95816, USA; Hawkesbury Institute for the Environment, Western Sydney University, Locked Bag 1797, Penrith, NSW 2751, Australia; Hawkesbury Institute for the Environment, Western Sydney University, Locked Bag 1797, Penrith, NSW 2751, Australia; Department of Natural and Physical Sciences, Shepherd University, 301 N King St, Shepherdstown, WV 25443, USA; Hawkesbury Institute for the Environment, Western Sydney University, Locked Bag 1797, Penrith, NSW 2751, Australia; Hawkesbury Institute for the Environment, Western Sydney University, Locked Bag 1797, Penrith, NSW 2751, Australia; Environment, CSIRO, Canberra 2601, ACT, Australia; Hawkesbury Institute for the Environment, Western Sydney University, Locked Bag 1797, Penrith, NSW 2751, Australia; College of Life Sciences, Zhejiang University, Hangzhou 310000, China

**Keywords:** model-data comparison, *Eucalyptus tereticornis,* temperature optimum, terrestrial biosphere models, forests

## Abstract

Understanding how canopy-scale photosynthesis responds to temperature is of paramount importance for realistic prediction of the likely impact of climate change on forest growth. The effects of temperature on leaf-scale photosynthesis have been extensively documented but data demonstrating the temperature response of canopy-scale photosynthesis are relatively rare, and the mechanisms that determine the response are not well quantified. Here, we compared leaf- and canopy-scale photosynthesis responses to temperature measured in a whole-tree chamber experiment and tested mechanisms that could explain the difference between leaf and crown scale temperature optima for photosynthesis. We hypothesized that (i) there is a large contribution of non-light saturated leaves to total crown photosynthesis, (ii) photosynthetic component processes vary vertically through the canopy following the gradient in incident light and (iii) seasonal temperature acclimation of photosynthetic biochemistry has a significant role in determining the overall temperature response of canopy photosynthesis. We tested these hypotheses using three models of canopy radiation interception and photosynthesis parameterized with leaf-level physiological data and estimates of canopy leaf area. Our results identified the influence of non-light saturated leaves as a key determinant of the lower temperature optimum of canopy photosynthesis, which reduced the temperature optimum of canopy photosynthesis by 6–8 °C compared with the leaf scale. Further, we demonstrate the importance of accounting for within-canopy variation and seasonal temperature acclimation of photosynthetic biochemistry in determining the magnitude of canopy photosynthesis. Overall, our study identifies key processes that need to be incorporated in terrestrial biosphere models to accurately predict temperature responses of whole-tree photosynthesis.

## Introduction

**Figure 1:**
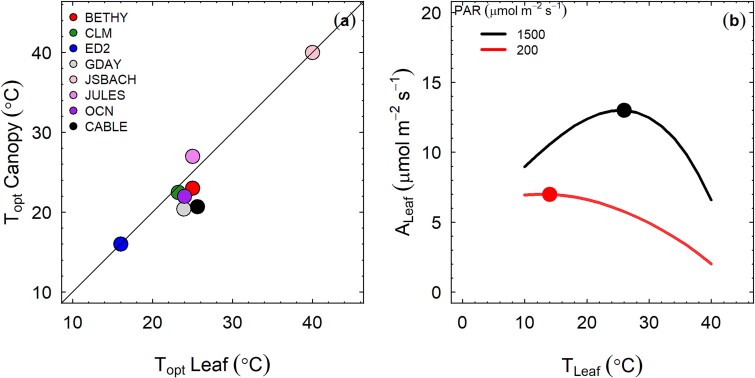
(a) The optimum temperature for leaf level and canopy level photosynthesis for eight models following Rogers et al. (2017). Model simulations were carried out under saturating irradiance level of 1500 μmol m^−2^ s^−1^ and vapour pressure deficit (VPD) of 1 kPa. The maximum carboxylation rate of Rubisco at 25 °C (V_cmax25_) was 60 μmol m^−2^ s^−1^. Leaf area index was fixed at 3. Data for models BETHY, CLM, ED2, GDAY, JSBACH, JULES and OCN were obtained from Rogers et al. (2017); the CABLE model was run independently following the same protocol. (b) The temperature responses predicted by the Farquhar et al. (1980) photosynthesis model at light levels of 1500 and 200 umol m^−2^ s^−1^. Model simulations were carried out under two irradiance levels; 1500 and 200 μmol m^−2^ s^−1^ and vapour pressure deficit (VPD) of 1 kPa. The maximum carboxylation rate of Rubisco at 25 °C (V_cmax25_) was 60 μmol m^−2^ s^−1^. The filled circles in panels (b) depicts the temperature optimum of leaf level photosynthesis.

Terrestrial Biosphere Models (TBMs) provide the foundation for predicting the fate of global forests in response to climate change (Zaehle et al. 2005, Medlyn et al. 2011, Rogers et al. 2017). Plant photosynthesis is one of the key processes in TBMs (Lombardozzi et al. 2015, Smith and Dukes 2017, Kumarathunge et al. 2019), meaning that robust representation of photosynthesis and its temperature response within TBMs is important for realistic prediction of the likely impact of climate change on forest growth, carbon sequestration and land–atmosphere feedback (Medlyn et al. 2015, Kala et al. 2016; D’Orangeville et al. 2018). Terrestrial Biosphere Models mechanistically represent plant photosynthesis and its response to temperature using parameterizations derived from data measured on individual leaves (Clark et al. 2011). The scaling of temperature responses from leaves to canopies determines the model projections of gross primary productivity of terrestrial ecosystems in response to warming (Huang et al. 2019). In many current TBMs, the temperature response of leaf- and canopy-scale photosynthesis are similar, with similar temperature optima (Rogers et al. 2017; Figure 1a). However, it is unclear whether this scaling of the photosynthetic temperature response from leaf to canopy in TBMs is correct as there is a dearth of empirical evidence on how leaf and canopy photosynthetic temperature responses compare. Further, the mechanisms that determine the photosynthetic temperature responses at canopy scale are not well understood, making it challenging to accurately represent canopy photosynthesis in TBMs.

The typical relationship between temperature and net photosynthesis at the leaf scale (*A*_net_) is parabolic, where *A*_net_ is maximal at the optimum temperature (*T*_optA_) and is lower at temperatures either below or above the *T*_optA_ (Battaglia et al. 1996, Gunderson et al. 2009, Crous et al. 2013, 2022). The *T*_optA_ of most C_3_ terrestrial plants ranges from 16 to 32 °C, and correlates well with the temperature of the growth environment (Kumarathunge et al. 2019, Crous et al. 2022). However, direct measurements of the temperature response of canopy scale photosynthesis are relatively rare. Several authors have used eddy covariance derived gross primary productivity data to quantify the temperature response of canopy photosynthesis (Niu et al. 2012; Tan et al. 2017; Huang et al. 2019). Using data from globally distributed eddy covariance sites, Niu et al. (2012) showed that the temperature response of net ecosystem exchange of different ecosystems followed a peak curve, with the optimum temperature ranging from 9 to 22 °C. The temperature response of net ecosystem exchange differs between cool- and warm-climate forests. In a high-elevation subalpine forest, the temperature optimum of net ecosystem exchange varied between 8 to 12 °C (Huxman et al. 2003). In comparison, Tan et al. (2017) reported that the temperature optimum for ecosystem scale photosynthesis for tropical forests ranged from 24 to 28 °C. These optimum temperatures for ecosystem-scale derived photosynthesis are often lower than the temperature optima for leaf-scale photosynthesis reported for the same species or plant functional type in a global analysis (Kumarathunge et al. 2019). For example, the temperature optimum for ecosystem scale photosynthesis of Arctic tundra reported by Huang et al. (2019) was at least 3 °C lower than the leaf level optimum reported by Kumarathunge et al. (2019). Similarly, temperature optima values reported by Tan et al. (2017) for Amazonian rain forest were 3 to 8 °C lower than the leaf level optima reported for the same forest by Kumarathunge et al. (2019). Crucially, the eddy covariance method has several limitations as it is not a direct measurement of canopy-scale gross primary productivity (Baldocchi 2003). Factors such as the temperature-dependence of soil respiration and water vapour pressure deficit may confound the resolution of temperature response functions of canopy scale photosynthesis derived from eddy covariance techniques (Bennett et al. 2021).

Whole-tree chambers (WTCs) overcome some limitations of the eddy covariance method by directly measuring crown scale photosynthesis (Medhurst et al. 2006; Barton et al. 2010). Whole-tree chambers directly measure the net flux of CO_2_ exchange between the entire tree crown and the surrounding air isolating the canopy gas exchange from CO_2_ evolved from the soil (Barton et al. 2012). Additionally, WTCs precisely control air temperature and relative humidity, which effectively minimizes the confounding effects of other temperature dependent factors such as VPD on the temperature response of canopy photosynthesis. Using data measured in a WTC experiment, Drake et al. (2016) reported that canopy-scale photosynthetic rates of *Eucalyptus tereticornis* trees measured at saturating incident light levels declined with increasing air temperature. There was no observable optimum temperature, but the data showed that this *T*_optA_ must have been lower than 16 °C (Drake et al. 2016). However, Duursma et al. (2014) reported that the optimum temperature for canopy-level photosynthesis of *Eucalyptus saligna* trees grown in WTCs was *c.* 22 °C which was lower than the leaf-level optimum temperature for photosynthesis (~25 °C) of the same trees.

There are several potential explanations for the observed difference between the temperature optima for photosynthesis at leaf- and canopy-scales. Firstly, it can be hypothesized that the lower temperature optimum of canopy photosynthesis, as observed in WTC experiments, is due to the contribution of non-light saturated leaves to the total canopy photosynthesis. Below light saturation, the rate of photosynthesis is related to the quantum yield, which declines monotonically with increasing temperature in C3 plants photosynthesis (Ehleringer and Björkman 1977, McMurtrie and Wang 1993). Figure 1b shows the temperature responses predicted by the Farquhar et al. (1980) photosynthesis model at light levels of 1500 and 200 μmol m^−2^ s^−1^. Due to self-shading and shading by neighbouring trees, much of the leaf area of a given tree canopy carries out photosynthesis at sub-saturating light levels (Ellsworth and Reich 1993), and hence the total canopy photosynthesis can be dominated by non-light saturated leaves, resulting in a lower temperature optimum.

Secondly, several studies provide evidence for significant differences in photosynthetic properties along the vertical axis of individual tree canopies following a gradient in light, including irradiance responses (Kull and Niinemets 1998; Kull and Kruijt 1999; Kull et al. 1999, Ranasinghe et al. 2015, Slot et al. 2018), photosynthetic biochemical properties (Carswell et al. 2000, Campany et al., 2016) and leaf maintenance respiration rates (Meir and Grace 2002; Weerasinghe et al. 2014). Hence, it can be hypothesized that the temperature optimum for photosynthesis is different between shade leaves and upper canopy sun-lit leaves of the same tree. If the contribution from shade leaves in a given canopy is significant, the temperature optimum for total canopy photosynthesis could be lower than the temperature optimum of upper canopy leaves. While the effect of lower maximum photosynthetic rates in the lower canopy has been explored (Kull and Kruijt 1999; Mercado et al. 2011; Mercado et al. 2009; Crous et al. 2021), the impact on the canopy temperature response has not been investigated (but see Hernandez et al 2020).

Additionally, it can be hypothesized that the observed decline in canopy photosynthesis with increasing temperature over several seasons, as observed, for example, in *E. tereticornis* trees (Drake et al. 2016) could be partly due to thermal acclimation of photosynthesis (Lin et al. 2012). Given the strong evidence for thermal acclimation of leaf photosynthesis (Medlyn et al. 2002b, Kattge and Knorr 2007; Way and Sage 2008; Crous et al. 2013, Lin et al. 2013, Way and Yamori 2014, Yamori et al. 2014, Yamaguchi et al. 2016, Crous et al. 2022), it is possible that longer-term effects of growth temperature on photosynthetic biochemistry could contribute to the overall temperature response of canopy photosynthesis constructed by aggregating measurements over time (e.g., Drake et al. 2016). Several studies have reported the consequences of incorporating thermal acclimation of photosynthesis into TBM for model predictions, showing improved ability to reproduce observed net ecosystem CO_2_ exchange (Lombardozzi et al. 2015, Smith et al. 2016, Mercado et al. 2018).

Given the need for robust quantification of photosynthetic temperature response at the canopy scale and its importance in predicting future forest and global carbon budgets, we designed this study to compare leaf- and canopy-scale temperature optima for photosynthesis. We used data from a WTC facility that measures whole-canopy photosynthesis at high temporal resolution across several seasons. We hypothesized that the observed difference between the temperature optimum for canopy and leaf photosynthesis is mainly due to the large contribution of non-light saturated leaves to total canopy photosynthesis. Additionally, we hypothesized that within-canopy gradients and seasonal temperature acclimation of photosynthetic biochemistry have a significant role in determining the temperature response of canopy photosynthesis. We tested these hypotheses using models of canopy radiation interception and photosynthesis, parameterized with leaf-level physiological data, estimates of canopy leaf area and tree dimensions. We applied the models under several different assumptions to predict whole-tree photosynthesis and evaluated the results against direct measurements of whole-tree photosynthetic flux. Our primary objective was to examine which mechanisms need to be incorporated into a model to accurately capture the temperature response of canopy photosynthesis.

## Materials and methods

### Whole tree chamber experiment

This study was conducted at the Hawkesbury Forest Experiment (33.62 S, 150.74 E), Richmond NSW, Australia. A detailed description of the site climatology and soil characteristics can be found in Crous et al. (2013), Aspinwall et al. (2016) and Drake et al. (2016). Briefly, the site mean annual temperature is 17 °C and the mean annual rainfall is 800 mm. The soils are low to moderately fertile sandy loam with relatively low organic matter content (~0.7%). The site contains 12 WTCs that each enclose a single tree rooted in soil. The WTCs are cylindrical in shape with a conical shaped top (9 m tall, 3.25 m in diameter and 53 m^3^ in volume). These chambers precisely control atmospheric CO_2_ concentration, air temperature, and relative humidity while measuring the net CO_2_ and water flux between the tree crown and atmosphere (Barton et al. 2010, Duursma et al. 2014, Drake et al. 2016). A detailed technical description of the WTCs can be found in Barton et al. (2010).

A single seedling of forest red gum (*Eucalyptus tereticornis* sp. tereticornis) was planted in the centre of each chamber on 12 March 2013. The air temperature of six chambers was set to track ambient temperature The remaining six chambers were set to track ambient temperature + 3 °C (i.e., the trees were warmed), but data from the elevated temperature treatment were not used in this analysis. Trees were watered fortnightly to keep them well-watered.

### Measurement of canopy net photosynthesis

Six months after the seedlings were planted, a suspended transparent plastic floor was installed at a height of 45 cm from the soil surface inside each chamber in order to isolate the canopy CO_2_ and H_2_O fluxes from fluxes evolved from the soil. At this time, trees in the ambient temperature treatment averaged 3 m tall, with stem diameter at 65 cm height of 2.4 cm. Average total canopy leaf area of the trees was 3.3 m^2^ (Drake et al. 2016). Whole-tree net CO_2_ flux was measured within each chamber at 15-min intervals (Barton et al. 2010) throughout the period starting in the austral spring from 13 September 2013 (floor installed) to 26 May 2014 (final harvest started). Flux data were corrected for chamber leaks and missing data (~6% of the total) and were gap filled using an artificial neural network method driven by tree size and weather variables. A detailed description of the data gap filling is available in Drake et al. (2016).

### Canopy photosynthesis simulation

We used three different leaf to canopy scaling approaches to test the stated hypotheses of this study. Firstly, we tested the ‘big-leaf’ approach (Table 1), where the tree crown was approximated as a single big leaf (Pury and Farquhar 1997). In this approach, photosynthetic capacity is assumed to decline through the canopy in proportion to the photosynthetically active radiation (PAR), allowing canopy photosynthesis to be calculated as a function of absorbed PAR (APAR) using the standard biochemical photosynthesis model of Farquhar et al. (1980) and the Medlyn et al. (2011) stomatal conductance model. Whole-canopy APAR was calculated as a function of incoming PAR and the fraction of incoming PAR (*f*PAR) absorbed by the tree leaf area. We calculated *f*PAR following Monsi and Saeki (1953) as


(1) \begin{equation*} fPAR=1-\exp \left(-k\ LAI\right) \end{equation*}

where *LAI* is the leaf area index and *k* is the canopy radiation extinction coefficient, here assumed to equal 0.5.

Secondly, we tested the ‘two-leaf’ approximation (Table 1) derived by Wang and Leuning (1998). In this approach, the tree crown is separated into a sunlit and shaded fraction, and the amount of PAR absorbed is calculated separately for the two canopy fractions. In the two-leaf model, the rate of decline of photosynthetic capacity with canopy depth is explicitly specified with an extinction coefficient on nitrogen in the canopy (*k*_n_ parameter in Table 2). Both the big-leaf and two-leaf models were implemented in R (https://github.com/mdekauwe/two_leaf_at_WTC).

**Table 1:** Model parameterization scenarios of tree canopy photosynthesis used in this study.

**Model**	**Parameterization scenario**	**Assumptions**
B. Single big leaf approximation	B1	Tree canopy assumed to behave as a single leaf. Leaf *V*_cmax_ and *J*_max_ are scaled to the canopy accounting for leaf area index and light extinction by assuming that photosynthetic capacity is proportional to incident PAR. An iterative scheme is used to solve the leaf energy balance (Medlyn et al. 2007). Horizontal homogeneity assumed.
	B2	B1 + Leaf-level *V*_cmax_ and *J*_max_ (at 25 °C) acclimate to seasonal changes in growth temperature.
T. Two big leaf approximation	T1	Tree canopy assumed to behave as two big leaves, sunlit and shaded. Leaf *V*_cmax_ and *J*_max_ are assumed constant through the canopy). An iterative scheme is used to solve the leaf energy balance (Medlyn et al. 2007). Horizontal homogeneity assumed.
	T2	T1 + Leaf *V*_cmax_ and *J*_max_ are scaled to the canopy accounting for leaf area index, canopy nitrogen and light extinction (Table 1, *k*_n_ and *k*_b_ parameters, respectively). Temperature response parameters of *V*_cmax_ and *J*_max_ are assumed constant through the canopy.
	T3	T2 + Leaf-level *V*_cmax_ and *J*_max_ (at 25 °C) acclimate to seasonal changes in growth temperature.
M. Multi-layer approximation	M1	Individual crowns are represented. Each crown is divided into six layers with 12 gridpoints, making 72 sub-volumes. Radiation extinction, self-shading and shading by neighbouring trees are included in simulations. An iterative scheme is used to solve the leaf energy balance (Medlyn et al. 2007). *V*_cmax_ and *J*_max_ are assumed constant through the six crown layers.
	M2	M1 + Leaf-level *V*_cmax_ and *J*_max_ (at 25 °C) decline with crown depth following the values given in Figure S2 available as Supplementary data at *Tree Physiology* Online. Temperature response parameters of *V*_cmax_ and *J*_max_ are assumed constant through the canopy.
	M3	M2 + Leaf-level *V*_cmax_ and *J*_max_ (at 25 °C) acclimate to seasonal changes in growth temperature.

Thirdly, we used a multilayer model, MAESTRA (https://maespa.github.io/), to simulate canopy photosynthesis. MAESTRA is an individual-tree based model of canopy CO_2_ flux and water balance, which calculates detailed radiation absorption, photosynthesis and transpiration of trees, growing individually or in a stand (Wang and Jarvis 1990; Duursma and Medlyn 2012). MAESTRA represents a forest canopy as an array of individual tree crowns. For each crown, spatial position (x and y co-ordinates), stem height, and several canopy metrics such as crown radius, height, leaf angle and one-sided leaf area can be specified (Duursma and Medlyn 2012). Each tree crown (target crown) is divided into several horizontal levels (typically six) and several grid points on each level, hence representing the target crown as an array of sub-volumes. For each grid point, MAESTRA calculates the sunlit and shaded fractions of leaf area, radiation transfer (PAR, NIR and long-wave), and direct and diffuse radiation fractions (Duursma and Medlyn 2012). The model calculates leaf net photosynthesis at each crown grid point using the standard biochemical photosynthesis model of Farquhar et al. (1980) and leaf-level stomatal conductance using the Medlyn et al. (2011) stomatal conductance model. Leaf temperature is calculated using an iterative approach following Medlyn et al. (2007). An exponential decline in photosynthetic capacity with canopy depth was approximated by specifying photosynthetic biochemical parameters *J*_max_ and *V*_cmax_ for each of six canopy layers. The baseline parameter values used in these model simulations are given in Table 1.

### Model parameterization

#### Meteorological data

**Figure 2:**
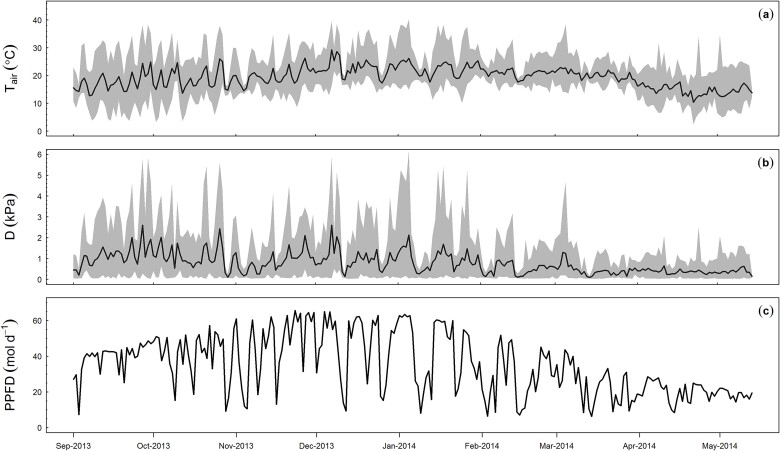
Meteorological data of the site during canopy photosynthesis measurement period: (a) daily mean air temperature (T_air_), (b) daily mean vapour pressure deficit (D), (c) daily integrated photosynthetically active radiation (PAR). In panels (a) and (b), the shaded area depicts the daily maximum and minimum values.

We measured half hourly incident PAR, air temperature (*T*_air_) and vapour pressure deficit (VPD) (Figure 2). Both *T*_air_ and VPD were directly measured inside the airspace of each chamber, while PAR was measured outside the chambers. A fixed ambient CO_2_ concentration of 400 µmol mol^−1^ (the WTC value) was assumed throughout the simulation period. The average daytime temperature and VPD during the canopy photosynthesis measurement period were 21.9 °C (range 13.6–31.4 °C) and 1.3 kPa (range 1.1–6.1 kPa), respectively.

#### Tree dimensions, canopy structure and leaf area

The total tree height, green crown height, height to crown base, tree diameter (at 65 cm above the soil surface) and crown radius in both north–south and east–west directions were measured fortnightly, throughout the canopy CO_2_ flux measurement period. Daily tree height increment for each tree was obtained by linearly interpolating the measured height (Drake et al. 2019). An individual-tree-specific allometric relationship was used to calculate the daily crown radius as a function of tree height. This model was developed and validated using the fortnightly measured crown width and tree height data (*r^2^ =* 0.99). Total crown leaf area of each tree was obtained from Drake et al. (2016). A detailed description of leaf area measurement and interpolation estimation can be found elsewhere (Barton et al. 2012, Drake et al. 2016).

The *E. tereticornis* crown was approximated as an upright cylinder and leaves were assumed to be randomly distributed within individual tree crowns. Leaf angles were assumed to be spherically distributed with five leaf angle classes. Crown leaf area density was assumed to be uniform and leaf clumping was not considered. Leaf reflectance and transmittance (400–700 nm waveband) values (Table 1) were obtained from measurements of *E. saligna* trees which were grown inside the same WTCs in a different experiment (Löw et al*.,* unpublished data).

#### Leaf scale physiology

We characterized the optimum temperature of leaf photosynthesis using two different datasets. In both cases we used only data from the six trees measured at ambient growth temperature treatment (the elevated temperature treatment was excluded). First, leaf-level light saturated net photosynthetic rate (*A*_sat_) was measured at prevailing ambient temperatures at monthly time intervals throughout the canopy photosynthesis measurement period by Aspinwall et al. (2016), using LI-6400XT portable photosynthesis systems (Li-Cor Inc., Lincoln, NE, USA). Measurements were conducted between 10:00 a.m. and 2:00 p.m. on recent fully expanded sun-lit leaves (two or three replicate leaves per tree) at a PAR of 1500 μmol m^−2^ s^−1^ and a flow rate of 500 μmol s^−1^. The relative humidity inside the leaf chamber was maintained between 60–80% and the sample CO_2_ concentration at 400 ± 5 p.p.m. The average leaf to air vapour pressure deficit during the measurements was 1.3 kPa (range 0.7–2.7 kPa) and the values were well aligned with the average VPD of canopy scale photosynthesis measurements (1.3 kPa, range 1.1–6.1 kPa). Measurements were made on two to three replicate leaves per tree and averaged across leaves to obtain a mean value for each tree. Following Battaglia et al. (1996) we characterized the temperature response of leaf scale photosynthesis by fitting a quadratic model (Eq. 2) to these *A*_sat_ measurements:


(2) \begin{equation*} {A}_{\mathrm{sat}}={A}_{\mathrm{opt}}-b{\left(T-{T}_{\mathrm{opt}\mathrm{A}}\right)}^2 \end{equation*}

where *A*_sat_ is the net photosynthetic rate (μmol m^−2^ s^−1^) at a given leaf temperature, *T* (°C)_,_*T*_optA_ is the temperature optimum for photosynthesis (°C) *A*_opt_ is the net photosynthetic rate at *T*_optA_, and the parameter *b* (unitless) describes the degree of curvature of the relationship. Throughout this work, we assume that leaf temperature was equivalent with the measured air temperature, which is consistent with the largely poikilothermic leaf temperatures observed for trees within this same WTC facility (Drake et al. 2020). We measured air temperature (*T* in Eq. 2) within each tree crown with duplicate, shielded, and aspirated thermistors (Dwyer RHP-2d2B, Michigan City, IN, USA and Mamac TE-702-A-12-A, Minneapolis, MN, USA) and assumed that canopy temperature is similar to the temperature of the surrounding air as there are strong evidence to support limited thermoregulation in leaf temperatures in Eucalyptus leaves (Drake et al. 2020). We estimated the parameters of Eq. 2 (i.e., *A*_opt_, *T*_optA_ and *b*) in a non-linear mixed model framework (Zuur et al. 2009) using the *nlme* function within the *nlme* package in R version 3.5.1. Replicate WTCs were included as random effects in the model.

The second set of data consisted of photosynthetic–CO_2_ response curves (A/C_i_). These were measured monthly on individual leaves at a standard leaf temperature of 25 °C between 10:00 a.m. and 2:00 p.m. on the same leaves used for *A*_sat_ measurements under similar light intensity, flow rate and humidity conditions as for measurements of *A*_sat_ by Aspinwall et al. (2016). In addition, A/C_i_ curves were measured on both upper canopy sun-lit leaves and lower canopy shade leaves (2–3 replicate leaves per tree) using the same method at a single time point (February 2014) by Campany et al. (2016). We used these A/C_i_ curves to estimate the maximum rate of Rubisco carboxylation (*V*_cmax_) and the maximum rate of electron transport for RuBP regeneration under saturating light (*J*_max_) using the FvCB model (Duursma 2015). Furthermore, A/C_i_ curves were measured on upper canopy sun-lit leaves at five different leaf temperatures between 25 and 42 °C in three seasons, spring 2013, summer and autumn 2014. We used these data to characterize the instantaneous temperature response of *A*_sat_*, V*_cmax_ and *J*_max._ The temperature response of *A*_sat_ was fitted using Eq. 2. The temperature responses of *V*_cmax_ and *J*_max_ were fitted using the peaked Arrhenius function (Johnson et al. 1942):


(3) \begin{equation*} {k}_{T_k}={k}_{25}\mathit{\exp}\left[\frac{E_a\left({T}_k-298.15\right)}{\left(298.15\ R\ {T}_k\right)}\right]\frac{1+\mathit{\exp}\left(\frac{298.15\Delta S-{H}_d}{\ 298.15\ R\ }\right)}{1+\mathit{\exp}\left(\frac{T_k\Delta S-{H}_d}{T_k\ R}\right)} \end{equation*}

where *k_Tk_* is the process rate (i.e., *V*_cmax_ or *J*_max_; $\mu$mol m^−2^ s^−1^) at a given temperature, *T*_k_ (K), *k_25_* is the process rate at 25 °C, *R* is the universal gas constant (8.314 J mol^−1^ K^−1^), and *E*_a_ (kJ mol^−1^) is the activation energy term that describes the exponential increase in enzyme activity with the increase in temperature, *H*_d_ (kJ mol^−1^) is the deactivation energy and *ΔS* (J mol^−1^ K^−1^) is the entropy term. To avoid over-parameterization, we assumed a fixed value of 200,000 J mol^−1^ for *H*_d_ in Eq. 3 for all trees (Dreyer et al. 2001, Medlyn et al. 2002a). We estimated the parameters of Eqn 3 (i.e., *k*_25_, *E*_a_ and *ΔS*) in a non-linear mixed model framework using replicate WTCs as random effects in the model.

Next, diurnally measured leaf-level photosynthesis measurements were used (see Drake et al. 2016 for a detailed description) to parameterize the stomatal conductance model. These measurements were conducted in five measurement campaigns from July 2013 to February 2014. Measurements were conducted at in-situ PAR, *T*_air_ and VPD conditions. We fitted the Medlyn et al. (2011) optimal stomatal conductance model to these data to estimate the stomatal conductance model parameters. We assumed zero minimum leaf conductance (*g*_0_) when fitting the stomatal conductance model (Duursma et al. 2018).

Finally, the estimated *V*_cmax_ and *J*_max_ for upper canopy sun-lit leaves and lower canopy shade leaves at a single time point (as described above) were used to calculate the within-crown decline in *V*_cmax_ and *J*_max_ in the multilayer model (Table 2). Here, we assumed that the first crown layer (crown depth = 1) is represented by the sun leaves and the sixth crown layer is represented by the shade leaves (crown depth = 6). We fitted an exponential relationship for *V*_cmax25_ and *J*_max25_ with the crown depth. Based on this relationship, *V*_cmax25_ and *J*_max25_ were calculated for each of the six crown layers (Figure S2a and b available as Supplementary data at *Tree Physiology* Online). In the two big-leaf model, the decline in photosynthetic capacity was specified using an extinction coefficient for nitrogen in the canopy, *k*_n_ = 0.3 (Table 2) following Lloyd et al. (1995).

**Table 2:** Parameter values used for simulating tree canopy photosynthesis in this study.

**Parameter**	**Definitions**	**Value**	**Units**
*V*_cmax25_	Maximum rate of Rubisco carboxylation	103.6^*^	μmol m^−2^ s^−1^
*J*_max25_	potential rate of electron transport	178.2^*^	μmol m^−2^ s^−1^
*E_a_*	Activation energy	*V*_cmax_: 59700 *J*_max_: 23800	J mol^−1^
*ΔS*	Entropy factor	*V*_cmax_: 634 *J*_max_: 627	J mol^−1^ K^−1^
*H_d_*	Deactivation energy	200,000	J mol^−1^
*α_J_*	Quantum yield of electron transport rate on an absorbed-PAR basis	0.26	μmol mol ^−1^
*θ*_J_	Curvature of electron transport rate relationship with APAR	0.57	unitless
*R*_*L*25_	Leaf respiration rate in light	0.9	μmol m^−2^ s^−1^
*Q_10_*	The proportional increase in R_day_ per 10 °C rise in temperature	2.0	unitless
*g_1_*	A parameter related to the marginal water cost of carbon	2.4	kPa^0.5^
Soil reflectance	% in three wavebands; PAR, NIR, thermal	0.1, 0.3, 0.05	unitless
Leaf reflectance	% in three wavebands; PAR, NIR, thermal	0.093, 0.34, 0.01	unitless
Leaf transmittance	% in three wavebands; PAR, NIR, thermal	0.082, 0.49, 0.05	unitless
*k_n_* (two-leaf model)	Extinction coefficient of nitrogen in the canopy	0.3	unitless
*k*_b_ (two leaf model)	Light extinction coefficient	0.5	unitless

^*^Values given are for the baseline model simulations.

These values were specified seasonally and for sun and shade leaves/different crown layers separately depending on the model simulation scenario given in Table 2.

#### Model-data comparison and data analysis

The 15-min canopy CO_2_ flux measurements were averaged over hourly intervals following Duursma et al. (2014). For this analysis, the elevated temperature treatment was excluded and only data of the six trees measured at ambient growth temperatures were considered. Hence, the analysis includes a total of ~ 37,000 hourly canopy photosynthesis observations measured in six chambers over 6 months, then three chambers for the final 3 months when a drought treatment was implemented on half of the chambers. First, the ability of the three canopy scaling methods to reproduce measured hourly rates of canopy photosynthesis was evaluated, using linear regression analysis. Secondly, the temperature response of modelled canopy photosynthesis was characterized using a subset of hourly photosynthetic rates at saturating incident light levels (PAR >1200 μmol m^−2^ s^−1^) and compared with the temperature response of observed canopy photosynthesis using fitted general additive models. We considered canopy photosynthesis at saturating light levels to avoid any confounding effects of PAR on the temperature response. This subset of data spans across the day from 9 a.m. to 4 p.m. with most data concentrated during 10 a.m. to 2 p.m., well aligned with the time of leaf scale measurements (Figure S1 available as Supplementary data at *Tree Physiology* Online). Additionally, it allowed us to directly compare the temperature response of photosynthesis at the canopy scale with the leaf-scale response as the leaf-level temperature response was always measured at saturating light levels. We used general additive models to characterize the temperature response of canopy photosynthesis. General additive models use a data driven approach which does not assume a prior functional relationship between the variables (Rigby and Stasinopoulos 2005). We used the *mgcv* package in R version 3.5.1 (R Development Core Team 2018) to fit the general additive models. We included replicate WTCs as a random effect in the general additive models. Here, we considered the fitted and observed temperature response functions to be significantly different if the 95% confidence intervals were non overlapping. We used linear regression analysis to test the goodness of fit (i) between the measured and modelled hourly photosynthetic rates including all hourly net photosynthetic rates and (ii) subset of hourly net photosynthetic rates at saturating incident light levels (PAR >1200 μmol m^−2^ s^−1^). The datasets and the analysis code used for the entire analysis is publicly available through https://bitbucket.org/Kumarathunge/canscal.

## Results

### Optimum temperature for photosynthesis (T_optA_)

**Figure 3:**
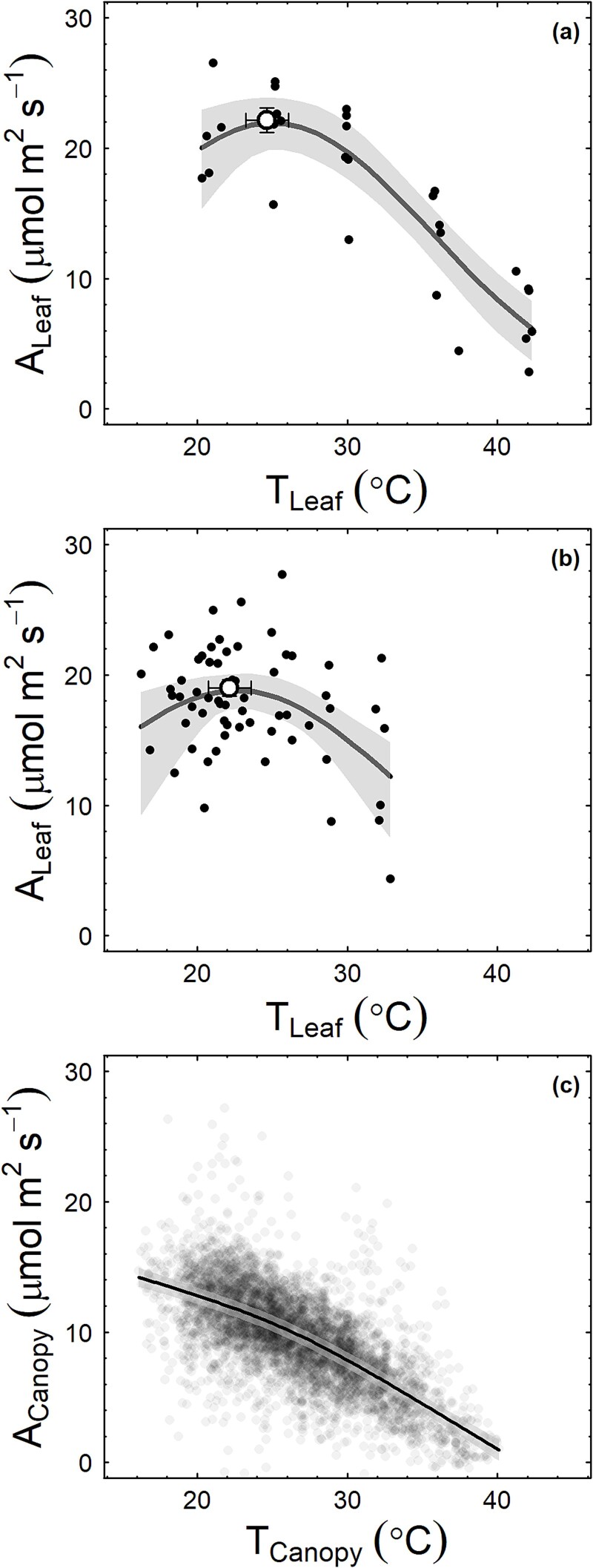
The temperature response of photosynthesis at different measurement scales: (a) instantaneous temperature response of leaf photosynthesis, (b) leaf photosynthesis measured at in-situ ambient temperatures across several seasons and (c) whole-canopy photosynthesis. Filled circles in panels (a) and (b), leaf-level net photosynthesis averaged across 2–3 replicate leaves on *E. tereticornis* trees (*n* = 6) grown under ambient growth temperatures in whole-tree chambers and in panel (c) hourly rates of net photosynthesis at incident PAR > 1200 *μ*mol m^−2^ s^−1^ expressed per unit crown leaf area (A_Canopy_) of the same trees (*n* = 6). Lines depict the fitted Eq. 2 in panels (a) and (b), general additive models in panel (c). The shaded area shows the 95% CI of predictions. T_canopy_ is the measured air temperature within each tree crown with duplicate, shielded, and aspirated thermistors (Dwyer RHP-2d2B, Michigan City, IN, USA and Mamac TE-702-A-12-A, Minneapolis, MN, USA). The circle with error bars (±1SE) in panels (a) and (b) depicts the temperature optimum of leaf level photosynthesis.

Both the leaf-scale instantaneous photosynthetic temperature response (Figure 3a) and net photosynthesis measured at ambient air temperatures (Figure 3b) showed an optimum temperature within the measurement temperature range. The optimum temperature for light-saturated leaf photosynthesis estimated using instantaneous measurements (24.7 ± 1.4 °C; Figure 3a) was higher than the optimum temperature estimated from measurements at ambient air temperatures (22.1 ± 1.4 °C; Figure 3b), but the 95% CIs overlapped. Both values were above the mean daytime temperature during the experiment, which was 19.4 °C (SD 3.6 °C). In contrast, measured high-light (PAR >1200 μmol m^−2^ s^−1^) canopy-scale net photosynthesis (*A*_Canopy_; μmol m^−2^ s^−1^) declined monotonically with increasing canopy temperature (*T*_Canopy_; °C; Figure 3c) and is lower than the leaf-scale net photosynthesis at any measurement temperature. The data did not show an optimum temperature for canopy photosynthesis within the measured air temperature range. *A*_Canopy_ approached zero when *T*_air_ was above 40 °C. Thus, the temperature optimum for the canopy photosynthesis of *E. tereticornis* trees in this experiment was at least 6–8 °C lower than the temperature optimum for light-saturated leaf net photosynthesis.

### Comparison of different leaf to canopy scaling models

**Figure 4:**
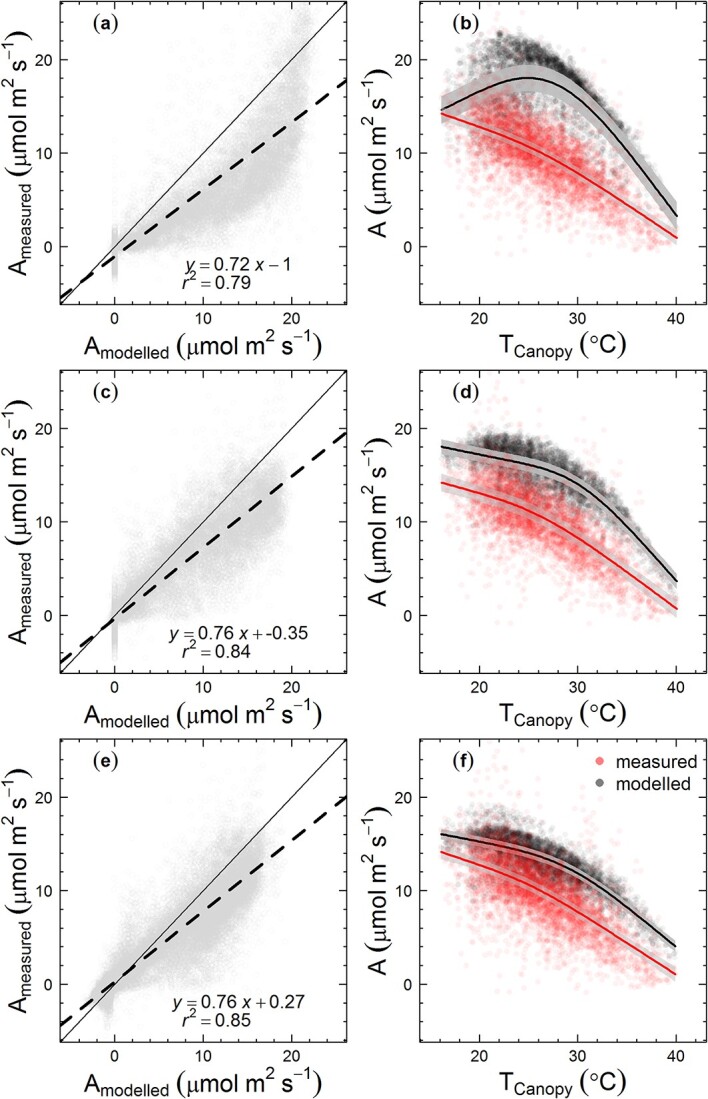
Measured vs modelled hourly rates of tree canopy net photosynthesis (A_measured_ and A_modelled_, respectively) and their temperature responses; (a) and (b) modelling the tree canopy as a single big leaf, (c) and (d) modelling the canopy as two big leaves (sunlit and shaded) and (e) and (f) modelling the tree canopy as multiple layers using MAESTRA (scenarios B1, T1, M1, respectively). Filled circles in panels (a), (c) and (e) depict daytime hourly net photosynthetic rates expressed per unit canopy leaf area at all PAR levels. Panels (b), (d) and (f) show hourly net photosynthetic rates at saturating light levels (PAR > 1200 μmol m^−2^ s^−1^), expressed per unit canopy leaf area. Dashed lines in panels (a), (c), (e) depict the fitted simple linear regression model and the continuous lines depict the 1:1 relationship. In panels (b), (d) and (f), lines depict the fitted general additive models with shaded areas showing the 95% CI of predictions. Results of the linear regression between the measured and modelled hourly photosynthetic rates at saturating incident light levels (PAR > 1200 μmol m^−2^ s^−1^) are given in Table 3.

We first modelled *E. tereticornis* canopy photosynthesis assuming that the tree canopy behaves as a single big-leaf (Scenario B1, Table 2 available as Supplementary data at *Tree Physiology* Online). This assumption was not able to accurately reproduce the measured canopy photosynthesis (Figure 4a). The model over-estimated canopy photosynthesis and had an incorrect temperature response for canopy photosynthesis at saturating PAR levels (Figure 4b). The modelled canopy photosynthesis showed an optimum temperature at ~25 °C, which was similar to the optimum temperature for leaf-scale photosynthesis estimated from instantaneous photosynthetic-temperature response measurements (Figure 4b).

Both the two big-leaf model and multilayer model (MAESTRA) with constant photosynthetic capacity (Scenarios T1, M1) showed a significant improvement in the predicted hourly canopy photosynthesis compared with the single big-leaf approximation (Figure 4c and f). The temperature response of modelled canopy photosynthesis in both the two big-leaf model and MAESTRA was much closer to the temperature response of measured canopy photosynthesis. The *r*^2^ values of the linear regression between the modelled and measured canopy photosynthesis at PAR >1200 μmol m^−2^ s^−1^ were 0.41 and 0.51 for two big-leaf model and MAESTRA, respectively. The RMSE value was decreased from 3.44 (single big leaf model) to 2.8 (two big-leaf model) and for MAESTRA, the RMSE was 2.53 (Table 3). However, for both the two big-leaf and multilayer models, there remained a systematic over-estimation in predicted canopy photosynthesis compared with the measurements at any given canopy temperature (Figure 4d).

**Table 3:** Results of the linear regression analysis between measured and modelled tree canopy photosynthesis at PAR >1200 μmol m^−2^ s^−1^. Values in parentheses are standard errors of parameter estimates.

**Model**	**Parameterization**	**Intercept**	**Slope**	***r*** ^**2**^	**RMSE**
Big leaf	B1—Baseline	4.6 (0.23)	0.33 (0.013)	0.12	3.44
	B2—Seasonal acclimation in *V*_cmax_ and *J*_max_	2.5 (0.22)	0.48 (0.014)	0.21	3.25
Two big-leaf	T1—Baseline	−1.9 (0.21)	0.79 (0.014)	0.41	2.80
	T2—Within canopy gradient in *V*_cmax_ and *J*_max_	−1.2 (0.21)	0.8 (0.015)	0.40	2.83
	T3—Within canopy gradient and seasonal temperature acclimation in *V*_cmax_ and *J*_max_	2.6 (0.17)	0.53 (0.012)	0.31	3.03
MAESTRA	M1—Baseline	−4 (0.19)	1.1 (0.014)	0.51	2.53
	M2—Within canopy gradient in *V*_cmax_ and *J*_max_	−3.5 (0.18)	1.1 (0.014)	0.52	2.51
	M3—Within canopy gradient and seasonal temperature acclimation in *V*_cmax_ and *J*_max_	−0.24 (0.15)	0.96 (0.014)	0.59	1.80

### Within-tree crown variation in photosynthetic biochemical parameters

**Figure 5:**
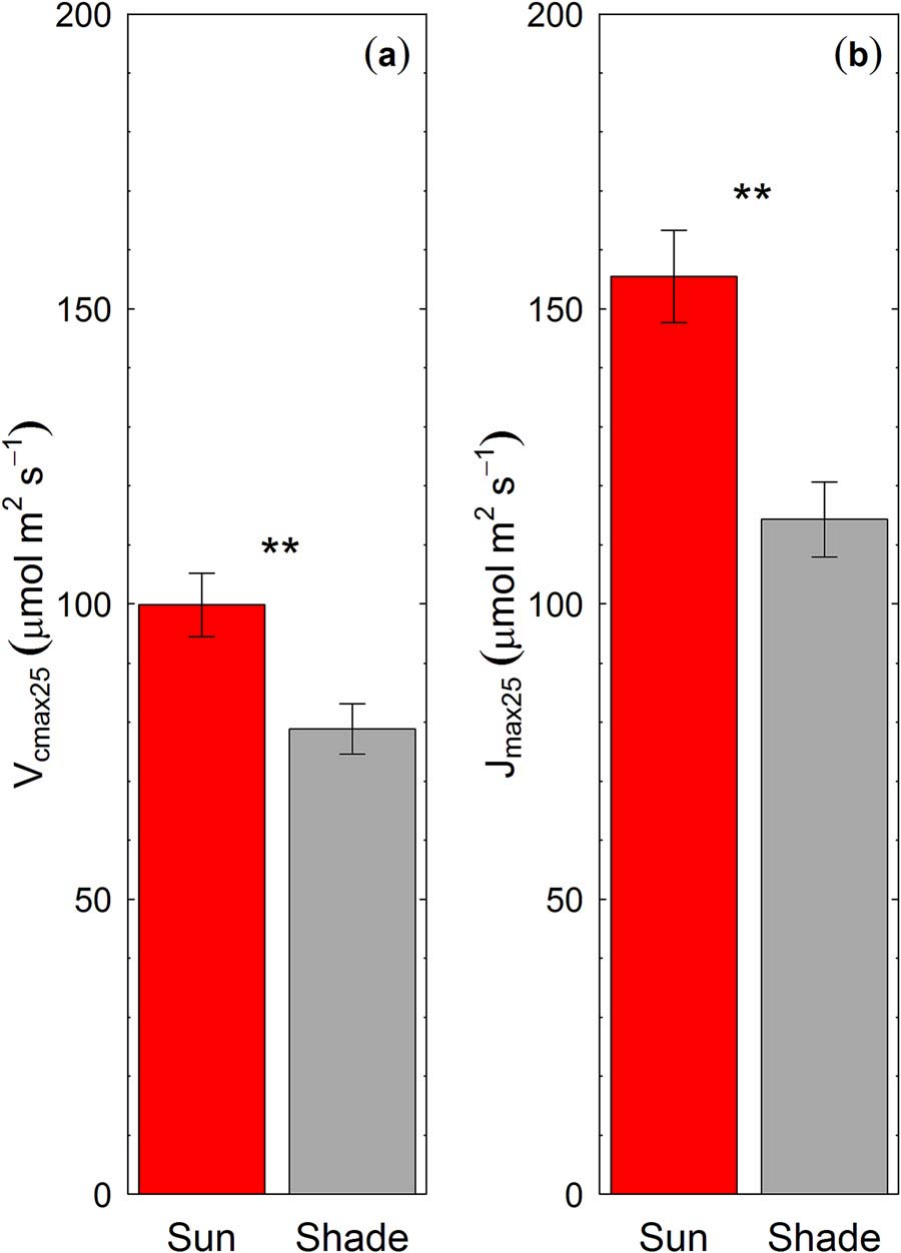
The maximum rate of carboxylation by the enzyme ribulose-1,5-bisphosphate carboxylase-oxygenase (V_cmax_; panel a) and the potential rate of electron transport (J_max_; panel b) of sun leaves and shade leaves of *E. tereticornis*. ^**^ denotes significant differences between sun and shade leaves at *α* = 0.001. Error bars represent ±1SE.

A clear divergence in both *V*_cmax_ and *J*_max_ was observed between sun and shade leaves (Figure 5). Both *V*_cmax_ and *J*_max_ were higher for sun leaves compared with shade leaves (Figure 5, ANOVA; *P* < 0.05). *V*_cmax25_ was 21% lower in shade leaves relative to the upper canopy sun leaves. Similarly, *J*_max25_ was 26% lower in shade leaves, similar to data from an independent study on the same species (Crous et al. 2021).

**Figure 6:**
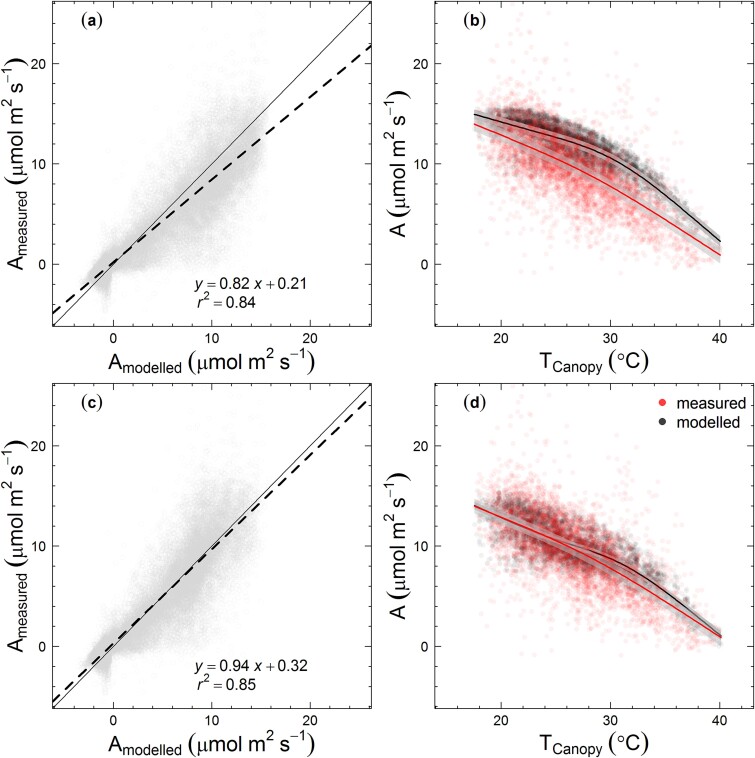
Measured vs modelled hourly rates of tree canopy net photosynthesis (A_measured_ and A_modelled_, respectively) and their temperature responses. Here, the multilayer crown model MAESTRA was used. In (a) and (b), an exponential decline of both *V*_cmax_ and *J*_max_ within the tree crown was assumed (scenario M2). In (c) and (d), both an exponential decline and seasonal temperature acclimation of *V*_cmax_ and *J*_max_ were assumed (scenario M3). Points in panels (a) and (c) show all hourly net photosynthetic rates expressed per unit canopy leaf area at all PAR levels; points in panels (b) and (d) show the hourly net photosynthetic rates for PPFD > 1200 μmol m^−2^ s^−1^, also expressed per unit canopy leaf area. In panels (a) and (c), dashed lines indicate the fitted simple linear regression model and continuous lines indicate the 1:1 line. In panels (b) and (d), lines depict the fitted general additive models with shaded areas showing the 95% prediction CI. Results of the linear regression between the measured and modelled hourly photosynthetic rates at saturating incident light levels (PAR > 1200 μmol m^−2^ s^−1^) are given in Table 3.

We implemented the observed within-crown variation in photosynthetic capacities in the two big-leaf model and MAESTRA, but this assumption did not significantly improve the predictions of the two big-leaf model (Table 3). However, in the multi-layer model (MAESTRA), it reduced the bias such that the slope of predicted vs. observed canopy photosynthesis was increased towards unity from 0.76 to 0.82. It did not improve the temperature response of canopy photosynthesis as model outputs did not show a significant change to the shape of the temperature response function compared with the baseline two big-leaf model and MAESTRA (Figure 6b). Neither the *r*^2^ nor RMSE of the two big-leaf and MAESTRA models were significantly changed (Table 3). There remained a systematic overestimation in the model compared with the measurements.

### Seasonal acclimation in photosynthetic biochemical parameters

Aspinwall et al. (2016) found strong evidence for acclimation of the maximum rate of carboxylation by the enzyme ribulose-1,5-bisphosphate carboxylase-oxygenase (*V*_cmax_) to the prevailing growth temperature. *V*_cmax_ measured at a standard temperature of 25 °C (*V*_cmax25_) showed a significant decreasing trend with increases in the preceding 30-day mean air temperature (*T*_growth_). Both parameters were significantly higher in winter compared with summer. The mean rate of decrease in *V*_cmax_ was 1.9 ± 0.6 μmol m^−2^ s^−1^ per °C increase in *T*_growth_ (Figure S3a available as Supplementary data at *Tree Physiology* Online). Similar to *V*_cmax_, the potential rate of electron transport measured at 25 °C (*J*_max25_) showed a significant decreasing trend with increasing *T*_growth_ (Figure S3b available as Supplementary data at *Tree Physiology* Online). The rate of decrease in *J*_max_, 3.7 ± 0.8 μmol m^−2^ s^−1^ per °C increase in *T*_growth_ was higher than that of *V*_cmax_. Implementation of seasonal variation in *V*_cmax_ and *J*_max25_ alone (without within crown variation in *V*_cmax_ and *J*_max25_) slightly improved the predictions such that the slope of predicted vs. observed canopy photosynthesis was increased towards unity from 0.76 to 0.82. (Figure S4 available as Supplementary data at *Tree Physiology* Online).

Next, we implemented both the within-crown gradient and seasonal acclimation in *V*_cmax_ and *J*_max_ in all three models (Scenarios B2, T3, M3, Table 2) by assigning both parameters monthly using leaf-level data from Aspinwall et al. (2016). Here, we assumed that the photosynthetic-temperature acclimation capacity was similar in leaves irrespective of the canopy position. This assumption significantly improved the predicted canopy photosynthesis only in the multi-layer model (Figure 6c), where the slope of the linear regression between measured and modelled canopy photosynthesis increased from 0.82 to approach the 1:1 line at 0.94. Also, the temperature responses of photosynthesis of measured and modelled canopy photosynthesis were not significantly different, as the 95% confidence intervals were overlapping (Figure 6d). The *r*^2^ values of the linear regression between the modelled and measured canopy photosynthesis at PAR > 1200 μmol m^−2^ s^−1^ increased from 0.52 (scenario M2) to 0.59 (scenario M3) and the RMSE decreased from 2.51 to 1.8 μmol m^−2^ s^−1^. In the two big-leaf model, implementation of a within-crown gradient and seasonal temperature acclimation in *V*_cmax_ and *J*_max_ did not significantly improve the predicted canopy photosynthesis compared with the multilayer model and the *r*^2^ values were lower and the RMSE was higher in the two big-leaf model under this assumption compared with MAESTRA (Table 3). Overall, our results identified both within-canopy spatial variation and seasonal temperature acclimation in photosynthetic biochemical parameters as important determinants of the temperature response of tree canopy photosynthesis.

## Discussion

In this study, we explored the temperature response of tree canopy photosynthesis using a model-data integration approach. We found that the observed lower temperature optimum for canopy photosynthesis than for leaf photosynthesis could be attributed to several factors: the large contribution of leaves operating at non-saturating light levels, the variation of photosynthetic capacity vertically through the canopy following the gradient in light and seasonal thermal acclimation of photosynthetic capacity to changing growth temperature. Further, we demonstrated that the big leaf model was not able to account for the reduction in the temperature optimum at the canopy scale, but the two big-leaf and multilayer approaches were able to predict the temperature response of canopy photosynthesis accurately. Overall, these results suggest that radiation extinction through the canopy layers as well as gradients in photosynthetic biochemical parameters within the canopy and thermal acclimation across seasons are the principal determinants of the temperature dependence of canopy photosynthesis.

Our study demonstrated significant improvement in predicted hourly canopy photosynthesis after accounting for irradiance extinction and leaf shading within the tree canopy (due to both self-shading and effects of neighbouring trees). This result provides a strong indication that often the canopy leaf area operates under light-limited conditions. Therefore, our data suggested that the observed differences in the temperature optimum for leaf and canopy photosynthesis is partly due to the large contribution of non-light saturated leaves to the total canopy photosynthesis, in support of our first hypothesis. Several previous studies have suggested that canopy scale photosynthesis is frequently dominated by non-light saturated leaves (Ellsworth and Reich 1993, McMurtrie and Wang 1993, Schäfer et al. 2003; Yang et al. 2019). At non-light-saturated conditions, photosynthetic rates of individual leaves are negatively affected by increasing photorespiration with temperature (Ehleringer and Björkman 1977), and hence the canopy photosynthesis rate tends to decrease with increasing temperature (McMurtrie and Wang 1993, Haxeltine and Prentice 1996, Drake et al. 2016). As a result, the temperature response of photosynthesis of leaves in lower canopy layers shows a monotonically declining relationship at non-saturating irradiance conditions even with high incident PAR at the top of the canopy. Additionally, leaf temperatures are likely to differ within the canopy, with shaded leaves operating at a lower temperature than sunlit leaves. This effect is accounted for in our multi-layer model, in which we dynamically calculate leaf temperatures from energy balance in each layer, and may help to explain the improved performance of the multi-layer model. However, here we expect this effect to be relatively small. Detailed measurements in a subsequent experiment with a related species (*E. parramattensis* instead of *E. tereticornis* studied here) indicated that leaf temperatures measured directly closely followed the air temperatures as measured by the thermistors within the crown (Drake et al. 2020). There were only modest deviations in T_leaf_ relative to T_air_ that were correlated with PPFD (e.g., 1–2 °C). Our results suggest that both single and two big-leaf (sunlit and shaded) approximations do not accurately predict the temperature response of canopy photosynthesis and provide evidence for the need to account for radiation extinction and shading within the tree canopy.

Our results showed a slight improvement in modelled canopy photosynthesis in the multi-layer model after accounting for the vertical canopy gradient in leaf-scale photosynthetic capacities, *V*_cmax_ and *J*_max_. In the two big-leaf model, this improvement was relatively minor in magnitude when compared with the improvement in modelled photosynthesis in multilayered MAESTRA. Many previous studies provide evidence for significant differences in photosynthetic parameters, including *V*_cmax_ and *J*_max_, along the vertical axis of individual tree canopies following a gradient in light (Kull et al. 1999, Carswell et al. 2000, Meir et al. 2002, Weerasinghe et al. 2014, Ranasinghe et al. 2015, Campany et al. 2016, Slot et al. 2018, Crous et al. 2021). Mercado et al. (2007) tested different vertical configurations of *V*_cmax_ in the Joint UK Land Environment Simulator (JULES) and reported that simulated photosynthetic carbon uptake was less sensitive to variation in *V*_cmax_ with canopy depth. As our results showed, if the majority of the canopy leaf area is operating under light-limited conditions, it is highly likely that the canopy photosynthesis is principally limited by light rather than *V*_cmax_. Alternatively, it can be hypothesized that the systematic over-estimation in the model compared with the measurements could potentially be due to the values for *V*_cmax_ and *J*_max_ for leaves at the lower canopy layers being assumed to be higher than the actual values. Because our data show a clear temporal trend in both *V*_cmax_ and *J*_max_ following seasonal changes in growth temperature (discussed later), the parameter values assumed under a multilayer modelling scenario alone do not represent the observed leaf-scale photosynthetic biochemistry. Note that we did not consider variation in the stomatal parameter *g*_1_ across different seasons and canopy depth as an explanation for differences between observed and modelled canopy photosynthesis, as we assumed constant *g*_1_ parameter throughout the canopy. There is little empirical evidence for seasonality in g1 either in our data, or in that from other Eucalyptus species grown at the same location in the WTCs (Barton et al. 2012) or in the field (Héroult et al. 2013; Gimeno et al. 2016). Data from Campany et al. (2016) do suggest that *g*_1_ of leaves in the lower canopy is higher than that in the leaves of upper canopy, but this difference would tend to increase the optimum temperature for photosynthesis of lower-canopy leaves (Lin et al. 2012) so does not help to explain the low canopy-scale optimum temperature for photosynthesis.

Our results showed a major improvement in the modelled canopy scale photosynthesis predictions by the multi-layer scheme after accounting for both a within-canopy gradient and seasonal temperature acclimation of *V*_cmax_ and *J*_max_ (i.e., supporting our second hypothesis). Under these assumptions, the model was able to predict canopy photosynthesis values that more closely matched the observations compared with the big leaf and two big-leaf models with the same assumptions. Additionally, this modelling scenario captures the temperature response of canopy photosynthesis accurately. Kumarathunge et al. (2019) clearly showed that acclimation of photosynthetic biochemical component processes to growth temperature was the principal driver of the leaf-scale photosynthetic temperature response. Previous studies on eucalypts also provide evidence for temporal variation in photosynthetic capacities following changes in growth temperature (Ghannoum et al. 2010, Crous et al. 2013, 2018, Lin et al. 2013, Drake et al. 2015, Aspinwall et al. 2016, 2017). These findings provide evidence for the need for systematic accounting for seasonal temperature acclimation in predicting canopy photosynthesis. Our results are further supported by several global scale studies which reported significant improvement in predictions of TBM after accounting for acclimation in photosynthetic biochemical parameters (Smith et al. 2016, Mercado et al. 2018).

In this study, we assumed that the photosynthetic-temperature acclimation capacity was similar in sun and shade leaves. Previous studies on photosynthetic-temperature acclimation have mainly focused on upper canopy sun-lit leaves (Crous et al. 2013, Aspinwall et al. 2016, Yamaguchi et al. 2016, Slot and Winter 2017). Constraining the photosynthetic acclimation capacity of leaves at different canopy depths is challenging as we lack a detailed understanding on how leaf scale photosynthetic-temperature response parameters (i. e. *E*_a_, *H*_d_ and ΔS) vary in leaves at different canopy positions. Nevertheless, our results indirectly suggest that the temperature response parameters of *V*_cmax_ and *J*_max_ (i.e., *E*_a_, *H*_d_ and Δ*S*) estimated at upper canopy sun-lit leaves sufficiently predict the temperature response of canopy photosynthesis.

Several TBM still use the ‘big-leaf’ assumption when scaling leaf photosynthesis to canopies (Amthor 1994, Rogers et al. 2017), while others represent sun and shade leaf area separately using the one-layered ‘two-big-leaves’ approximation (Pury and Farquhar 1997, Dai et al. 2004). Results of this study provided evidence for the potential need of multilayer approaches in modelling canopy photosynthesis (Bonan and Patton 2021). This study disentangled the processes that need to be incorporated in TBM to accurately predict temperature responses of tree canopy photosynthesis. These findings on leaf to canopy scaling of photosynthetic temperature response derived in this study may prove useful in quantifying tree and forest canopy photosynthesis and improve the predictions of photosynthesis in TBM. 

## Supplementary Material

SI_R2_tpae135

## Data Availability

The datasets and the analysis code used for the entire analysis is publicly available through https://bitbucket.org/Kumarathunge/canscal.
