# Supplementary material for: Photosynthetic temperature responses in leaves and canopies: why temperature optima may disagree at different scales
Source: Tree Physiol. 2024 Oct 17;44(11):tpae135. doi: 10.1093/treephys/tpae135 (PMC11585359; doi:10.1093/treephys/tpae135)
Supplement: SI_R2_tpae135 [file si_r2_tpae135.docx]

***Tree Physiology* Supporting Information**

Article title: Photosynthetic temperature responses in leaves and canopies: why temperature optima may disagree at different scales?

Dushan P. Kumarathunge, Belinda E. Medlyn, John E. Drake, Martin G. De Kauwe, Mark G. Tjoelker, Michael J. Aspinwall, Craig V. M. Barton, Courtney E. Campany, Kristine Y. Crous, Jinyan Yang, Mingkai Jiang


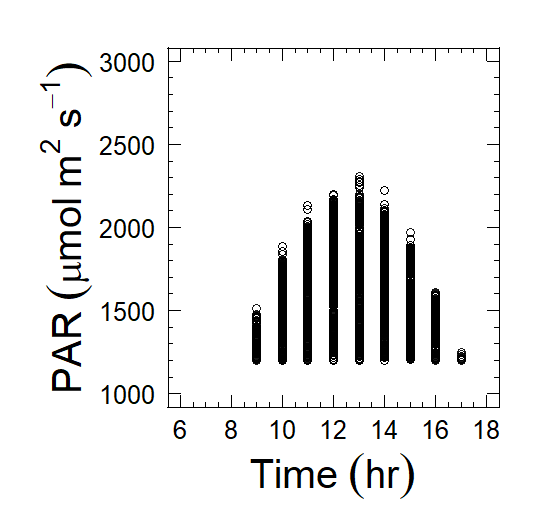


Figure S1. Distribution of PAR (>1200 µmol m^-2^s^-1^ across time of the day during measurement period.


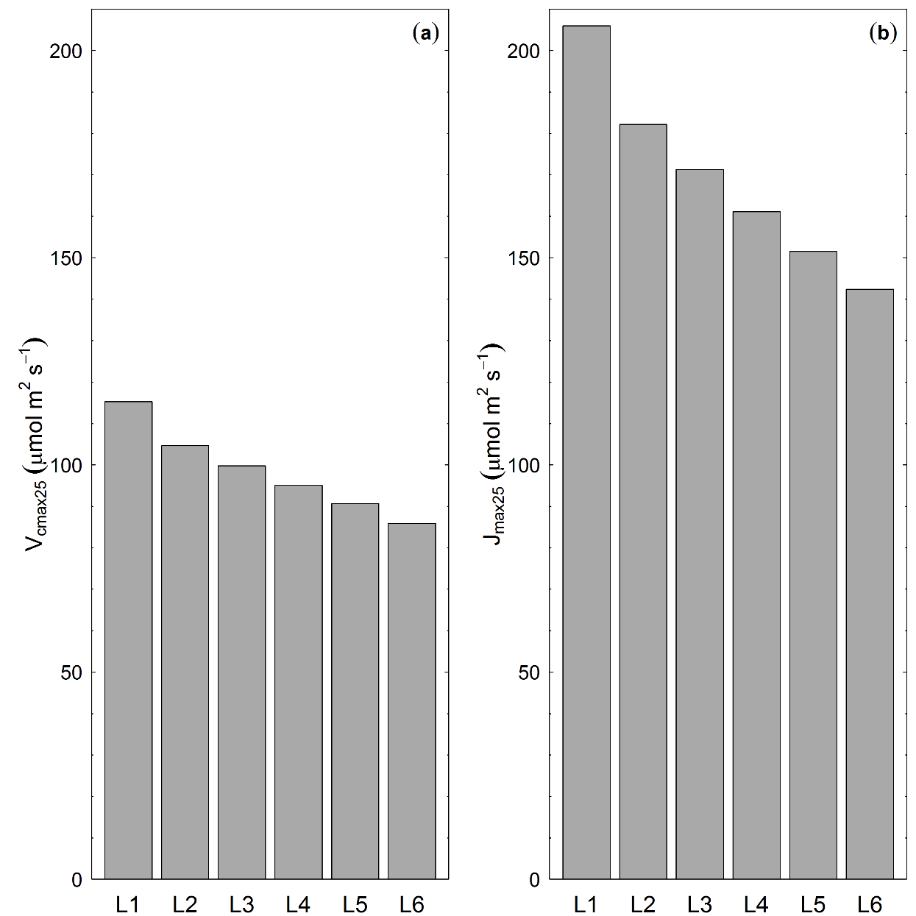


Figure S2. The maximum rate of carboxylation by the enzyme ribulose-1,5-bisphosphate carboxylase-oxygenase (*V*_cmax_; panel a) and the potential rate of electron transport (*J*_max_; panel b) at a standard temperature of 25°C of six tree crown layers (L1-L6) of *E. tereticornis* that were used in multilayer model. L1 represents the upper canopy sun lit layer (crown depth=1) and L6 is the lowest crown layer (crown depth=6). Values are log-linearly interpolated from sunlit and shaded leaf values from Campany et al. (2018).


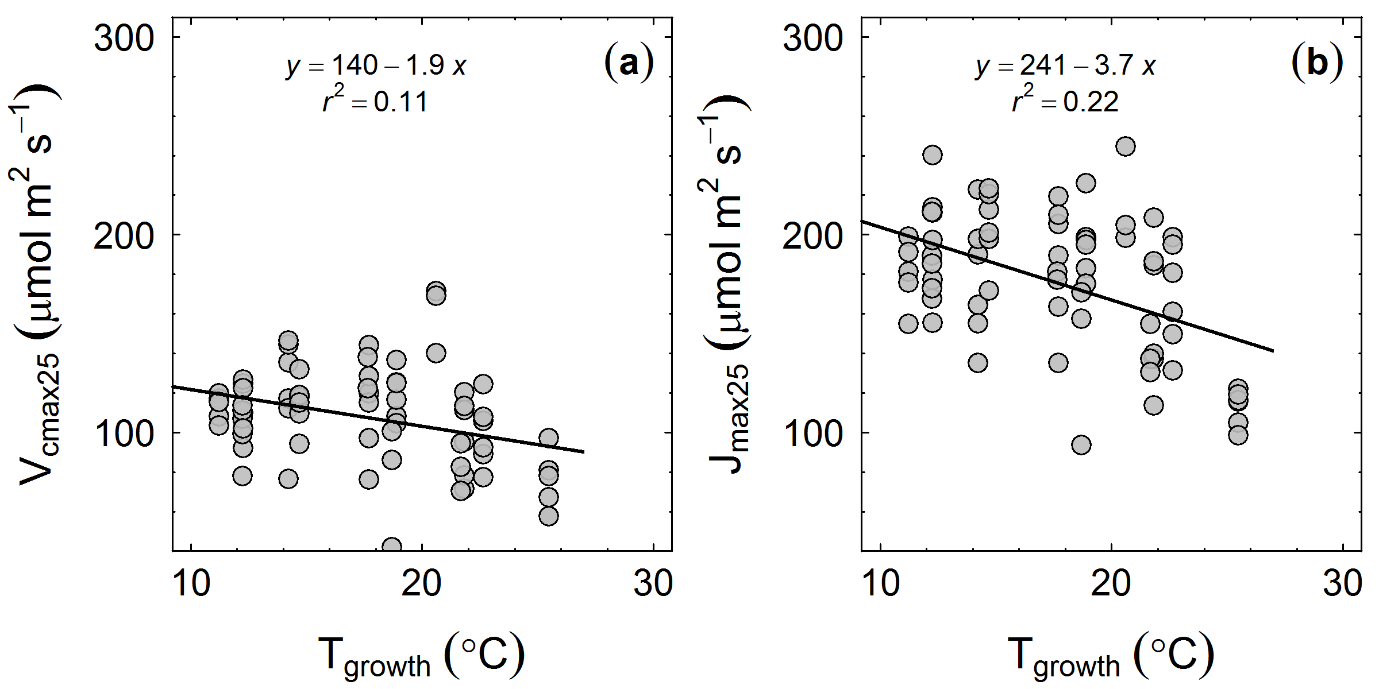


Figure S3. Seasonal acclimation of the maximum rate of carboxylation by the enzyme ribulose-1,5-bisphosphate carboxylase-oxygenase (*V*_cmax_; panel a) and the potential rate of electron transport (*J*_max_; panel b). Filled circles in panels (a) and (b) depict *V*_cmax_ and *J*_max_ values at 25 °C (*V*_cmax25_ and *J*_max25_ respectively, n=3) measured on replicate leaves (n=3) Lines depict the fitted least-squares regression models. T_growth_ is the mean air temperature for the 30 days prior to gas exchange measurements. Error bars represent ±1SE. Data from Aspinwall et al. (2016).


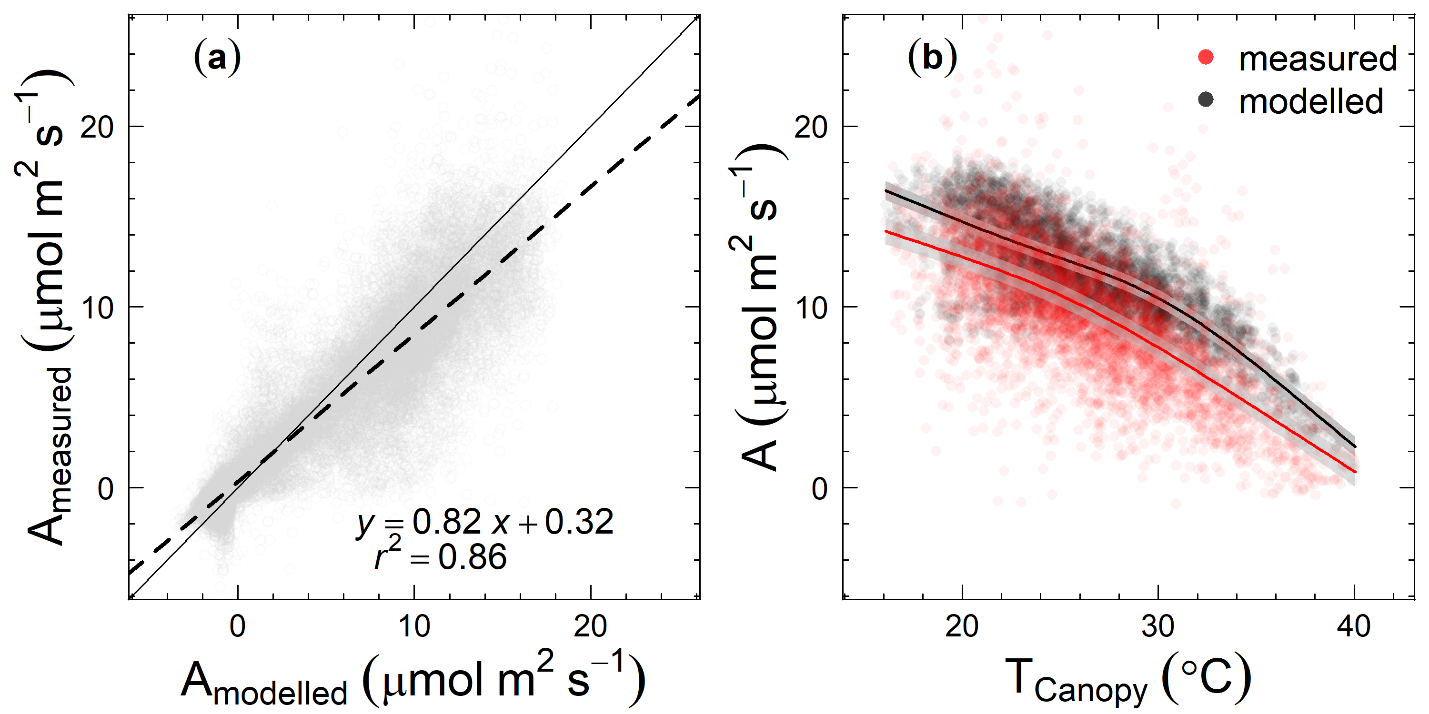


Figure S4. Measured vs modelled hourly rates of tree canopy net photosynthesis (A_measured_ and A_modelled_ respectively) and their temperature responses. Here, the multilayer crown model MAESTRA was used. *V*_cmax_ and *J*_max_ are assumed constant through the six crown layers and seasonal temperature acclimation of *V*_cmax_ and *J*_max_ were assumed (scenario M1+ seasonal temperature acclimation of *V*_cmax_ and *J*_max_). Points in panels (a) show all hourly net photosynthetic rates expressed per unit canopy leaf area at all PAR levels; points in panels (b) and show the hourly net photosynthetic rates for PPFD >1200 µmol m^-2^ s^-1^, also expressed per unit canopy leaf area. In panels (a), dashed lines indicate the fitted simple linear regression model and continuous lines indicate the 1:1 line. In panels (b), lines depict the fitted general additive models with shaded areas showing the 95% prediction CI.
